# Supplementary material for: Connectome-driven neural inventory of a complete visual system
Source: Nature. 2025 Mar 26;641(8065):1225–37. doi: 10.1038/s41586-025-08746-0 (PMC12119369; doi:10.1038/s41586-025-08746-0)
Supplement: Supplementary file 2 — Reporting Summary [file 41586_2025_8746_MOESM2_ESM.pdf]

nature portfolio

Corresponding author(s): Stuart Berg, Gerald M. Rubin, Michael B. Reiser

Last updated by author(s): Dec 5, 2024

Reporting Summary

Nature Portfolio wishes to improve the reproducibility of the work that we publish. This form provides structure for consistency and transparency in reporting. For further information on Nature Portfolio policies, see our Editorial Policies and the Editorial Policy Checklist.

Statistics

For all statistical analyses, confirm that the following items are present in the figure legend, table legend, main text, or Methods section.

n/a	Confirmed	
☒	☐	The exact sample size (*n*) for each experimental group/condition, given as a discrete number and unit of measurement
☒	☐	A statement on whether measurements were taken from distinct samples or whether the same sample was measured repeatedly
☐	☒	The statistical test(s) used AND whether they are one- or two-sided<br>*Only common tests should be described solely by name; describe more complex techniques in the Methods section.*
☒	☐	A description of all covariates tested
☒	☐	A description of any assumptions or corrections, such as tests of normality and adjustment for multiple comparisons
☐	☒	A full description of the statistical parameters including central tendency (e.g. means) or other basic estimates (e.g. regression coefficient) AND variation (e.g. standard deviation) or associated estimates of uncertainty (e.g. confidence intervals)
☐	☒	For null hypothesis testing, the test statistic (e.g. *F*, *t*, *r*) with confidence intervals, effect sizes, degrees of freedom and *P* value noted<br>*Give P values as exact values whenever suitable.*
☒	☐	For Bayesian analysis, information on the choice of priors and Markov chain Monte Carlo settings
☒	☐	For hierarchical and complex designs, identification of the appropriate level for tests and full reporting of outcomes
☒	☐	Estimates of effect sizes (e.g. Cohen's *d*, Pearson's *r*), indicating how they were calculated

Our web collection on statistics for biologists contains articles on many of the points above.

Software and code

Policy information about availability of computer code

Data collection	This project relied on code at many levels for data collection, reconstruction, curation, etc. All details are extensively documented in the Methods and the software tools used at each step are referenced.
Data analysis	The python code that replicates our analysis and data visualization is available via GitHub: https://github.com/reiserlab/male-drosophila-visual-system-connectome-Python-3.10 (or higher) is required with further dependencies detailed in GitHub repository. We make use of: python 3.12.5, navis 1.7, neuprint-python 0.4.26, snakemake 8.20.3, pymupdf 1.24.10, cloud-volume 10.4, google-cloud-storage 2.18.2 fastcluster 1.2.6, numpy 1.26, alphashape 1.3.1, kneed 0.8.5. The Code Availability section of the methods explains this repository. Extensive documentation is available in this repository to help readers replciate our figures.

For manuscripts utilizing custom algorithms or software that are central to the research but not yet described in published literature, software must be made available to editors and reviewers. We strongly encourage code deposition in a community repository (e.g. GitHub). See the Nature Portfolio guidelines for submitting code & software for further information.

Data

Policy information about availability of data

All manuscripts must include a data availability statement. This statement should provide the following information, where applicable:

- Accession codes, unique identifiers, or web links for publicly available datasets
- A description of any restrictions on data availability
- For clinical datasets or third party data, please ensure that the statement adheres to our policy

All data is publicly available.

Data Availability statement:
The connectome data is directly accessible via the neuPrint database server: https://neuprint.janelia.org/?dataset=optic-lobe:v1.1

The Cell Type Explorer web resource is available at: https://reiserlab.github.io/male-drosophila-visual-system-connectome/index.html
and can also be downloaded as a zip file from DOI:10.5281/zenodo.10891950

The SWC skeleton is available through neuPrint+ (web interface) by clicking the bodyId in the skeleton viewer, which will provide a download button. For bulk downloads, the meshes of neurons, ROI boundaries and skeletons are provided as Google Cloud Storage buckets at gs://flyem-optic-lobe. In our shared code (see Code availability) we provide programmatic access to these files. For example, the skeletons and meshes of individual neurons are accessible through the OLNeuron class. The example Jupyter notebook: https://github.com/reiserlab/male-drosophila-visual-system-connectome-code/blob/main/src/python-bootcamp/access_skeleton_and_mesh.ipynb shows how to store the skeleton as a *.swc file and the mesh as a Wavefront *.obj file.

The LM-EM transformation vectors from our EM sample to the JRC2018M template brain are at https://figshare.com/s/e17528e5e2c44ba78b5d, also stored at gs://flyem-optic-lobe/transforms/MaleCNS_JRC2018M.h5. In that file, the "dfield" transformation vectors map points from EM space to LM template space, and "invdfield" vectors map points in the opposite direction.

Images of split-GAL4 driver lines are available at https://splitgal4.janelia.org/cgi-bin/splitgal4.cgi.

Research involving human participants, their data, or biological material

Policy information about studies with human participants or human data. See also policy information about sex, gender (identity/presentation), and sexual orientation and race, ethnicity and racism.

Reporting on sex and gender	n/a
Reporting on race, ethnicity, or other socially relevant groupings	n/a
Population characteristics	n/a
Recruitment	n/a
Ethics oversight	n/a

Note that full information on the approval of the study protocol must also be provided in the manuscript.

Field-specific reporting

Please select the one below that is the best fit for your research. If you are not sure, read the appropriate sections before making your selection.

☒ Life sciences ☐ Behavioural & social sciences ☐ Ecological, evolutionary & environmental sciences

For a reference copy of the document with all sections, see nature.com/documents/nr-reporting-summary-flat.pdf

Life sciences study design

All studies must disclose on these points even when the disclosure is negative.

Sample size	The EM dataset reported in this study represents the visual system of a single male fly. Over 50000 individual neurons were reconstructed. Light microscopy (LM) images of genetic driver lines include at least two (typically more) images per driver line.
Data exclusions	No data were excluded.
Replication	The EM dataset is currently the only reconstruction of the visual system of a male Drosophila melanogaster and therefore cannot be independently replicated at this time. However, many of the observed cell morphologies are supported by LM data and, for some cell types,

prior EM data. LM images of expression patterns of driver lines were examined for at least two flies. Since the original submission we have compared all the cell types to the FlyWire dataset of a female brain and have matched 98% of the cell types between the data sets.

Randomization: N/A

Blinding: N/A

Reporting for specific materials, systems and methods

We require information from authors about some types of materials, experimental systems and methods used in many studies. Here, indicate whether each material, system or method listed is relevant to your study. If you are not sure if a list item applies to your research, read the appropriate section before selecting a response.

Materials & experimental systems

n/a	Involved in the study	
☐	☒	Antibodies
☒	☐	Eukaryotic cell lines
☒	☐	Palaeontology and archaeology
☐	☒	Animals and other organisms
☒	☐	Clinical data
☒	☐	Dual use research of concern
☒	☐	Plants

Methods

n/a	Involved in the study	
☒	☐	ChIP-seq
☒	☐	Flow cytometry
☒	☐	MRI-based neuroimaging

Antibodies

Antibodies used: We used antibodies against epitope tags (HA, V5 and FLAG) and GFP to detect transgenically expressed reporter constructs and a widely used monoclonal antibody against Brp (clone: mAb Nc82) as a general neuropile label. We use well-established protocols that have been used in many studies, with additional details described in our previous studies: Wu et. Al. 2016, Nern et al 2015, Meissner et al 2023, Meissner et al 2024, and availabe: https://www.janelia.org/project-team/flylight/protocols

Validation: All antibodies used in this study have been extensively used and validated previously (https://www.janelia.org/project-team/flylight/protocols)

Animals and other research organisms

Policy information about studies involving animals; ARRIVE guidelines recommended for reporting animal research, and Sex and Gender in Research

Laboratory animals: All experiments used laboratory strains of Drosophila melanogaster. This study reports a collection of transgenic fly lines (split-AGL4 lines), genotypes of which are listed in Supplementary Table 6. Images are of 1-8 day old flies.

Wild animals: No wild animals were used.

Reporting on sex: The EM dataset is of a single male fly. For light microscopy images, fly sex is included in the image metadata (available with the images at https://splitgal4.janelia.org/cgi-bin/splitgal4.cgi).

Field-collected samples: No field collected samples were used.

Ethics oversight: No ethics oversight required.

Note that full information on the approval of the study protocol must also be provided in the manuscript.

Plants

Seed stocks: n/a

Novel plant genotypes: n/a

Authentication: n/a
