# Supplementary material for: Connectome-driven neural inventory of a complete visual system
Source: Nature. 2025 Mar 26;641(8065):1225–37. doi: 10.1038/s41586-025-08746-0 (PMC12119369; doi:10.1038/s41586-025-08746-0)
Supplement: Supplementary file 4 — Supplementary Tables 1–7 and a guide to the tables. [file 41586_2025_8746_MOESM4_ESM.zip › Supplementary Tables Guide.pdf]

Supplementary Table 1: Cell type names, counts, and neurotransmitter predictions for the inventory of visual neurons. The table lists all of the right-side instances (majority of cells, instance labels end with "_R") followed by the left-side instances (instance label end with "_L"). Related to Fig. 1, Supplementary Fig. 1.

Supplementary Table 2: Fraction of synaptic connections outside the primary visual system neuropils. Related to Extended Data Fig. 2.

Supplementary Table 3: The unique identifiers (bodyId) and assigned coordinates for the 15 columnar cell types of Fig. 2a. The column-by-column pale vs. yellow designation is also provided. There are a small number of double assignments—pairs of the same cell type found in the same columnar coordinate, and these are separated by a comma. Related to Fig. 3, Extended Data Fig. 5.

Supplementary Table 4: The matching of T4 neurons to Mi1 neurons for creating columns in LOP. Related to Extended Data Fig. 6.

Supplementary Table 5: Neurotransmitter ground truth (for training) and experimental validation data. Related to Fig. 4.

Supplementary Table 6: List of split-GAL4 lines and the cell types labeled by these lines. Related to Fig. 7, Extended Data Fig. 14, 15.

Supplementary Table 7: Preliminary matches for the (male) optic lobe cell types to the annotated cell types in the (female) FlyWire dataset. Cell type names, counts, and neurotransmitter predictions from both datasets are listed, together with links to visualizations of the matched cell types. The neurotransmitter predictions that cannot be compared are greyed out, while the mismatched predictions are colored in red. Further details in Methods. Related to Extended Data Fig. 16.
